# Nutritional status of healthcare professionals in primary health and social care

**DOI:** 10.1371/journal.pone.0325422

**Published:** 2025-06-04

**Authors:** Svetlana Plyassovskaya, Xeniya Mkhitaryan, Yelena Pozdnyakova

**Affiliations:** 1 School of Public Health and Biomedicine, Karaganda Medical University, Karaganda, Republic of Kazakhstan; 2 Department of Physiology, Karaganda Medical University, Karaganda, Republic of Kazakhstan; 3 Department of Biomedicine, Karaganda Medical University, Karaganda, Republic of Kazakhstan; The University of Adelaide - North Terrace Campus: The University of Adelaide, AUSTRALIA

## Abstract

Balanced nutrition is crucial for healthcare workers’ health and performance. Despite extensive knowledge of healthy eating, they face organizational and behavioral barriers leading to suboptimal diets. This study aimed to compare anthropometric status and dietary intake among male physicians, female physicians, and nurses in primary healthcare, and to identify deficiencies or excesses of key nutrients. A descriptive cross-sectional study was conducted in six clinics in Karaganda (2023–2024), involving 202 participants (22 male physicians, 67 female physicians, and 113 nurses). Body composition (weight, fat percentage, muscle mass, bone mass, metabolic age) was assessed via Tanita BC-418MA bioelectrical impedance. Dietary intake was measured using repeated 24-hour recalls for proteins, fats, carbohydrates, fiber, vitamins, and minerals. Statistical analysis included nonparametric methods (Kruskal–Wallis, Dunn’s test) and the Wilcoxon signed-rank test. Results showed insufficient intake of proteins, carbohydrates, dietary fiber, calcium, potassium, magnesium, phosphorus, and vitamins B₁, B₂, PP, and C. Sodium intake was excessive in all groups, whereas iron deficiency was observed only in women. Male physicians had higher body weight, muscle mass, and bone mass than female groups, but there were no significant differences in BMI, metabolic age, or overall dietary composition among the groups. Female physicians consumed slightly more vitamin C than nurses. In conclusion, gender and professional role minimally influenced dietary patterns among primary healthcare workers. Despite mostly normal BMI values, the pervasive lack of essential nutrients, coupled with high sodium and fat consumption, emphasizes the need for institutional measures such as structured schedules and healthier food options.

## Introduction

Modern nutritional research consistently confirms the critical importance of a balanced diet in maintaining health and preventing a wide range of diseases [1,2]. Adequate intake of dietary nutrients, including proteins, fats, carbohydrates, vitamins, and minerals, is fundamentally necessary not only for sustaining normal physiological functions but also for reducing the risk of chronic diseases associated with metabolic disorders [3,4].

Unbalanced or insufficient nutrition significantly contributes to the increasing prevalence of obesity, type 2 diabetes, and cardiovascular diseases, while excessive consumption of certain foods – especially those rich in refined carbohydrates and trans fats – further exacerbates this trend [5,6].

The 2023 report “The State of Food Security and Nutrition in the World” emphasizes that access to healthy nutrition remains unattainable for billions of people. In 2023, approximately 2.33 billion people worldwide experienced moderate to severe food insecurity. These statistics highlight the importance of ensuring a diet that meets both energy and nutrient requirements as a key factor in reducing the prevalence of chronic non-communicable diseases, increasing life expectancy, and promoting active longevity [7]. Equally important is the quality of the diet, which should include sufficient intake of vegetables, fruits, dietary fiber, and essential vitamins and minerals [8].

In the context of Central Asian countries – Uzbekistan, Kyrgyzstan, Tajikistan, and Kazakhstan – the issue of balanced nutrition is complicated by a combination of climatic, historical, and socioeconomic factors [9,10]. The region has a distinctly continental climate, characterized by hot, dry summers and extremely cold winters with sharp temperature fluctuations [11,12]. These conditions have historically led to a predominance of high-calorie foods rich in animal fats and carbohydrates, providing energy during periods of summer heat and warmth during harsh winter frosts. Traditional livestock farming has also played a significant role, supplying an abundance of meat and dairy products (such as lamb, beef, horse meat, and fermented dairy beverages), which have become a cultural norm in local diets [13].

Kazakhstan, as one of the key countries in Central Asia, follows this dietary pattern. According to reports from the Ministry of Health of the Republic of Kazakhstan, a significant portion of the population consumes excessive amounts of table salt, sugars, and animal fats while not meeting the recommended intake of vegetables, fruits, fiber-rich grains, and legumes. In rural areas, there is a seasonal deficiency of quality protein sources and fresh produce during autumn and winter, whereas urban areas exhibit a “Westernized” dietary model characterized by high consumption of processed and refined foods [14].

The dietary habits of healthcare professionals play a crucial role not only in maintaining their own health and work capacity but also in shaping their professional credibility among patients [15]. However, even within the medical community – where there is extensive knowledge of healthy eating principles—various eating disorders and nutrient imbalances are frequently observed [16].

An analysis of factors influencing the nutrition of physicians and nursing staff shows that the problem is exacerbated by both objective organizational constraints (short meal breaks, limited food choices) and the personal behavioral habits of medical workers [17]. Several qualitative studies emphasize that skipping regular meals is often linked to high workloads and the desire to devote as much time as possible to patients [18]. For example, one study on the impact of structured meal breaks found that strictly scheduled mealtimes help maintain stable energy levels and cognitive function in physicians throughout the day [19]. However, such initiatives often remain isolated efforts and fail to develop into systematic policies.

The issue of access to healthy food is further complicated by the fact that hospitals and clinics predominantly offer high-calorie, prepackaged foods with excessive fat and sugar content [20]. The availability of healthier meal options is limited not only by their relatively high cost but also by the lack of well-organized food service infrastructure, particularly during evening and night shifts. These barriers force physicians and nurses to eat “on the go,” rely on snacks, and consequently develop unhealthy dietary habits [21].

Socioeconomic and cultural factors also play a role. Some studies suggest that young professionals (residents, interns) could develop healthier eating habits if provided with opportunities for self-cooking and guidance on meal planning [22]. At the same time, long-term professional experience and age may contribute to the accumulation of “unhealthy” habits, although research findings on this issue remain contradictory [23].

The influence of gender and professional status (physician or nurse) on dietary behavior remains an open question. Some studies suggest that female healthcare professionals are more likely to skip meals due to dual workloads – both professional and domestic – whereas other authors emphasize the greater impact of general organizational factors [24].

Consumption of certain food products (e.g., processed meats) raises additional concerns regarding potential health risks. For example, a study on the effects of nitrosamines among healthcare workers on night shifts found that even at relatively low levels of carcinogenic compounds in the diet, significant differences were observed between different personnel groups. These findings suggest that professional factors and personal dietary preferences interact, shaping a heterogeneous nutritional profile among healthcare professionals [25].

Thus, current scientific data indicate several common nutritional issues among physicians and nursing staff: excessive intake of fats and salt, insufficient intake of protein and vitamins, frequent meal skipping during working hours, and the absence of systemic institutional measures to support healthy eating in medical institutions [26–28]. However, it remains unclear to what extent the dietary habits of physicians and nurses differ in everyday practice and which factors – demographic or professional – play a more decisive role in determining their intake levels of key macro- and micronutrients.

In this context, the present study aims to conduct a detailed comparative analysis of actual food consumption among primary healthcare workers, taking into account differences between physicians and nursing staff. Examining the levels of macronutrient, micronutrient, and dietary behaviors will help identify potential vulnerabilities in their diets that may have long-term consequences for the health and professional efficiency of medical workers. The results obtained will serve as a scientific basis for developing targeted recommendations and future intervention programs aimed at optimizing nutrition in healthcare institutions.

## Materials and methods

### Study design

This study was conducted as a descriptive cross-sectional study. Data collection was carried out by the authors themselves from October 9, 2023, to October 1, 2024. Prior to the study, all authors involved in data collection received standardized training on the use of equipment and administration of dietary recall protocols. The research focused on assessing the anthropometric and physiological parameters, as well as the actual dietary intake of physicians and nurses from six clinics in Karaganda, Kazakhstan. Data collection was performed through questionnaires and laboratory assessments (bioelectrical impedance analysis) without any intervention in the habitual diet of the participants.

### Study population

The study included healthcare professionals (physicians and nurses) from the following six clinics in Central Kazakhstan (Karaganda):

1. Clinic of Occupational Diseases, NAO “KMU”
2. Clinic “Merei”
3. Clinic “Miras”
4. Polyclinic No. 4
5. Polyclinic No. 3
6. Polyclinic No. 8

The study included medical professionals (physicians and nurses) who met the following inclusion criteria: they had a medical education, were between 20 and 60 years old, were employed full-time or part-time, and had a minimum work experience of 3–5 years.

Participants were excluded from the study if they were pregnant or lactating, or had severe oncological or psychiatric disorders. Detailed medical diagnoses (e.g., diabetes mellitus, cardiovascular or renal disease) were not collected in accordance with institutional data‑protection rules; therefore, personnel with such conditions were not excluded.

The study included four groups of healthcare professionals: male physicians, female physicians, female nurses, and male nurses. A total of 202 participants were enrolled. The male physician group consisted of 22 individuals, the female physician group included 67 individuals, and the female nurse group comprised 113 individuals. The male nurse group was not represented in the final study, as only four male nurses were employed in the participating clinics, which was insufficient for statistical analysis.

Only four male nurses (3.4% of the 117 nurses employed in the six clinics) met the inclusion criteria. Although they consented to participate, the subgroup size was insufficient for statistical analysis; consequently, male nurses were excluded from group comparisons and are reported descriptively only.

### Ethical considerations

Before enrolment every participant received a written and oral explanation of study aims, procedures, potential risks/benefits and confidentiality safeguards, and then signed an informed‑consent form. The study protocol was approved by the Ethics Committee of Karaganda Medical University (Protocol No. 2, dated September 18, 2023). All procedures conducted in this study adhered to the ethical principles outlined in the Declaration of Helsinki (1975) and its subsequent revisions.

### Stages of the study

#### Bioelectrical impedance analysis.

Bioelectrical impedance analysis measurements were conducted using the Tanita body composition analyzer (Tanita BC-418MA, Tanita Corporation, Japan), which allowed for the assessment of the following parameters:

- Body Weight
- Fat Mass (FM)
- Muscle Mass
- Bone Mineral Content
- Basal Metabolic Rate (BMR)
- Body Fat Percentage (% BF)
- Body Mass Index (BMI)

Prior to data collection, Tanita BC‑418MA weight readings were cross‑checked against a hospital‑certified mechanical scale in 20 volunteers (mean difference 0.12 kg; p = 0.42). Height was measured with a Harpenden stadiometer, and BMI calculated accordingly. Impedance parameters were used as per manufacturer calibration, with daily self‑tests confirming device stability.

#### Body density.

Body density was calculated using the Durnin & Womersley formula. The calculation followed this algorithm:

1. The sum of skinfold thicknesses was calculated as follows: (subscapular skinfold + triceps skinfold + biceps skinfold + abdominal skinfold).
2. Body density was then determined using the formulas:

For men: 1.162−(0.063×log10(Sum of skinfolds)) = body density.

For women: 1.154−(0.067×log10(Sum of skinfolds)) = body density.

Fat tissue thickness was measured using a caliper (KEC-100–1-I model, TVES, Russia) at four sites: subscapular, triceps, biceps, and abdominal (suprailiac) skinfolds.

#### Metabolic age calculation.

Metabolic age was determined based on the basal metabolic rate (BMR) obtained through bioelectrical impedance analysis and standard reference data for different age groups. The algorithm included: • Consideration of chronological age and sex.

- Comparison of actual BMR with reference (average) BMR values for specific age groups recommended by the manufacturer of the Tanita analyzers.
- Determination of metabolic age based on the category within which the BMR value fell (with possible interpolation for intermediate values).

#### Dietary intake assessment.

Dietary intake analysis was conducted using the 24-hour recall method (three interview sessions). The questionnaire included information on meal timing, meal preparation and consumption, description of dishes and ingredients, cooking methods, and portion size and quantity of food consumed (reference to the questionnaire). The collected data were used to assess the daily energy value of the diet, as well as the content of macronutrients (proteins, fats, and carbohydrates), vitamins, and minerals.

Certain methodological rules were followed during the survey. Interviews were evenly distributed across all days of the week. Holidays were generally excluded from the survey. In practice, it was difficult to conduct interviews on Saturdays or Sundays, so interviews were conducted from Monday to Thursday. The survey was usually conducted in the morning or early afternoon, asking about meals chronologically from the first to the last meal of the previous day, covering the period from 00:00–24:00. The survey started with the first food or drink consumed in the morning, covering the entire daily intake from morning to morning over 24 hours. The interviews were conducted in a relaxed environment, in a quiet place, without the presence of outsiders or other respondents (S1 Appendix).

The chemical composition and energy value of the diet were analyzed using the software program «Program for calculating the nutritional and biological value of the average daily diet» [29]. Various normative sources were considered, including the World Health Organization (WHO), the European Food Safety Authority (EFSA), and the Institute of Medicine (IOM). The IOM (Institute of Medicine, USA) standards were used as reference values. These recommendations are widely applied in clinical practice and public health, establishing optimal intake levels for various population groups [30,31].

### Statistical processing

All collected data were analyzed using GraphPad Prism 8.0. Descriptive statistics were calculated, including median, interquartile range (IQR), minimum and maximum values, and overall range. Normality of distribution was assessed using the Anderson-Darling, D’Agostino-Pearson, Shapiro-Wilk, and Kolmogorov-Smirnov tests. In most cases, the distribution of variables did not meet normality criteria (p < 0.05), which justified the use of nonparametric methods.

To compare the median nutrient intake values obtained from the analysis of the chemical composition of the average daily diet with the recommended intake levels, statistical analysis was conducted using the Wilcoxon signed-rank test. This nonparametric test was used to assess differences between observed values and reference levels, allowing for the identification of statistically significant deviations from the norm.

For each variable, the median values, variance, and sum of positive and negative ranks, and p-values (two-tailed test) were calculated. The significance level was set at α = 0.05.

To compare three groups, the Kruskal-Wallis test was applied with a significance level of α = 0.05. Dunn’s multiple comparison test with a correction for multiple comparisons at a significance level of α = 0.0017 was used to clarify differences between groups.

## Results

### Anthropometric and associated body parameters

#### Chronological age.

The median age in the male physician group was 41.00 years (32.00–56.00), in the female physician group – 40.00 years (32.00–56.00), and in the female nurse group – 38.00 years (29.00–53.50) (age recorded in completed years). The minimum age was observed in the female nurse group (25 years), while the maximum was recorded in both female physician and female nurse groups (65 years). Accordingly, the age range was 32.00, 37.00, and 40.00 years for male physicians, female physicians, and female nurses, respectively (Table 1).

**Table 1:** Chronological and metabolic age indicators.

Group	Chronological age	Metabolic age
	Me (25%–75%)	Me (25%–75%)
**Male physicians**	41.00 (32.00–56.00)	39.00 (25.00–53.00)
**Female physicians**	40.00 (32.00–56.00)	36.00 (30.00–54.25)
**Female nurses**	38.00 (29.00–53.50)	36.00 (26.00–51.50)

**Fig 1:**
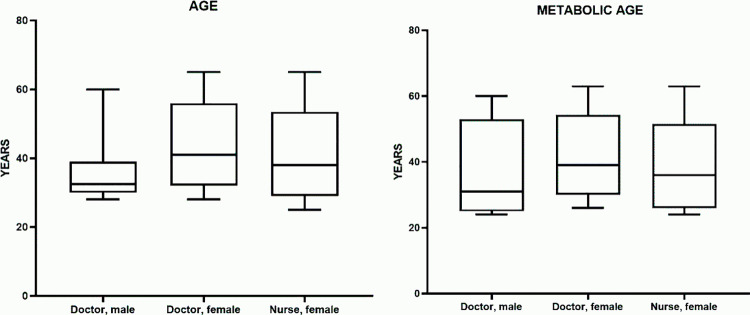
Chronological and metabolic age in the studied groups.

Comparison between gender groups showed that the median chronological age in female physicians was approximately 7.8% lower than in male physicians, while in female nurses, it was 8.5% lower. The differences between the “male physicians” and “female physicians” groups were statistically insignificant (p = 0.154). The comparison between “male physicians” and “female nurses” revealed statistically significant differences (p = 0.011). When comparing female physicians and female nurses, the median chronological age differed by only 0.8%; however, the difference was statistically insignificant (p = 0.599) (Fig 1).

Thus, a comparison of the three groups using the nonparametric Kruskal–Wallis test revealed no statistically significant differences between male and female physicians, as well as between female physicians and female nurses. However, statistically significant differences were found between male physicians and female nurses (with male physicians having a higher average value).

#### Metabolic age.

The median metabolic age in the male physician group was 39.00 years (25.00–53.00), in the female physician group – 36.00 years (30.00–54.25), and in the female nurse group – 36.00 years (26.00–51.50). The minimum metabolic age was observed in the male physician and female nurse groups (24 years), while the maximum was recorded in all three groups at 63 years. Accordingly, the range of values was 36.00, 37.00, and 39.00 years for male physicians, female physicians, and female nurses, respectively (Table 1).

A comparison between gender groups showed that the median metabolic age in female physicians was approximately 5.6% higher than in male physicians; however, this difference was not statistically significant (p = 0.1857). The comparison between male physicians and female nurses did not reveal changes in the median. When comparing female physicians and female nurses, the median metabolic age in nurses was 5.3% lower, but this difference was also statistically insignificant (p = 0.1532) (Fig 1).

A comparison of the three groups using the nonparametric Kruskal–Wallis test revealed no statistically significant differences between male and female physicians, as well as between female physicians and female nurses, or between male physicians and female nurses. In other words, there were no significant differences in metabolic age among the three studied groups.

All three groups demonstrated a “younger” metabolic age compared to chronological age. The difference in favor of metabolic age was the largest in male physicians (~32 years), slightly lower in female physicians (25 years), and in female nurses (26.5 years). Despite the fact that the male physician group had the highest median chronological age (68.3 years), they also exhibited a relatively “young” metabolic age (36 years). This significant difference may be associated with body composition characteristics (a higher proportion of muscle mass, a relatively lower proportion of fat tissue), as well as potential influences of individual health factors, lifestyle, and physical activity.

Female participants (physicians and nurses) had similar median chronological ages (63 years in physicians and 62.5 years in nurses) and metabolic ages (38 vs. 36, respectively). However, female physicians had a slightly smaller “gap” between their chronological and physiological ages (25 years), whereas nurses had a gap of about 26.5 years. Statistically, no significant difference in metabolic age was found between the female groups, indicating approximately similar body composition and basal metabolism levels on average.

#### Weight.

The median body weight in the male physician group70.60 kg (63.50–80.05), female physician group –63.00 kg (58.40–71.90), and infemale nurse group – 62.5062.50 kg (54.30–71.30). The minimum body weight was observed in the female nurse group (54.30 kg), whereas the maximum was recorded in the female physician group (110.30 kg). Accordingly, the data range was 67.90, 61.60, and 63.30 for male physicians, female physicians, and female nurses, respectively (Table 2).

**Table 2:** Weight and body mass index (BMI) indicators in the studied groups.

Group	Body weight (kg)	BMI (kg/m^2^)
	Me (25%–75%)	Me (25%–75%))
**Male physicians**	70.60 (63.50–80.05)	24.70 (21.50–25.60)
**Female physicians**	63.00 (58.40–71.90)	24.25 (21.40–27.35)
**Female nurses**	62.50 (54.30–71.30)	24.25 (20.48–28.10)

**Fig 2:**
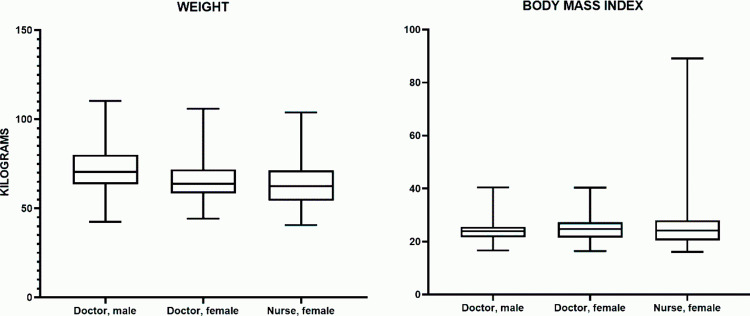
Body weight and BMI in the studied groups.

Comparison between gender groups showed that the median body weight in female physicians was approximately 7.7% lower than in male physicians, while in female nurses, it was 8.5% lower. The differences between the “male physicians” and “female physicians” groups were statistically insignificant (p = 0.154). The comparison between “male physicians” and “female nurses” revealed statistically significant differences (p = 0.011) (Fig 2).

Thus, a comparison of the three groups using the nonparametric Kruskal–Wallis test revealed no statistically significant differences between male and female physicians, as well as between female physicians and female nurses. Statistically significant differences were observed only between male physicians and female nurses (with male physicians having a higher average value).

#### Body mass index (BMI).

The median Body mass index (BMI) in the male physician group was 24.70 kg/m^2^ (21.50–25.60), in the female physician group – 24.25 kg/m^2^ (21.40–27.35), and in the female nurse group – 24.25 kg/m^2^ (20.48–28.10). The minimum BMI was observed in the female nurse group (16.10 kg/m^2^), while the maximum BMI was recorded in the female nurse group (89.20 kg/m^2^). Accordingly, the range of values was 23.80, 23.90, and 73.10 kg/m^2^ for male physicians, female physicians, and female nurses, respectively (Table 2).

A comparison of these indicators between groups showed that the median BMI in female physicians was approximately 3.3% higher than in male physicians. However, the difference between the “male physicians” and “female physicians” groups was statistically insignificant (p > 0.9999). The comparison between male physicians and female nurses revealed a difference of approximately 1.5% (with a higher median in female nurses), but this difference was also not statistically significant (p > 0.9999).

When comparing female physicians and female nurses, the median BMI differed by about 1.8%, but these differences also did not reach statistical significance (p > 0.9999) (Fig 2). Comparison between groups using the Kruskal–Wallis nonparametric test, followed by Dunn’s post hoc test, did not reveal statistically significant differences in pairwise comparisons (p > 0.9999). This indicates no significant differences in average BMI values between male physicians, female physicians, and female nurses.

### Body density indicators

The median body density (Me) in the male physician group was 1.029524 g/cm^3^ (1.030287–1.044287 g/cm^3^), in the female physician group – 1.029524 g/cm^3^ (1.017808–1.035024 g/cm^3^), and in the female nurse group – 1.028542 g/cm^3^ (1.016187–1.029861 g/cm^3^). Thus, the highest median body density was recorded in male physicians; however, the interquartile range in the female physician group was wider, suggesting greater variability in body density within this group. (Table 3).

**Table 3:** Body density, fat percentage, muscle mass, bone mass, and fat mass in the studied groups.

Indicator	Male physicians	Female physicians	Female nurses
	Me (25%–75%)	Me (25%–75%)	Me (25%–75%)
**Body density (g/cm**^3^)	1.029 (1.030–1.044)	1.029 (1.017–1.035)	1.028 (1.016–1.029)
**Fat percentage %**	26.33 (24.08–30.46)	30.85 (27.97–36.30)	32.47 (30.67–37.11)
**Muscle mass (kg)**	54.20 (49.68–57.65)	43.25 (39.90–45.43)	42.00 (39.80–44.70)
**Bone mass (kg)**	2.900 (2.800–3.000)	2.300 (2.100–2.400)	2.300 (2.100–2.400)
**Fat mass (kg)**	14.50 (8.45–23.65)	19.20 (12.80–27.05)	17.90 (9.70–26.10)

**Fig 3:**
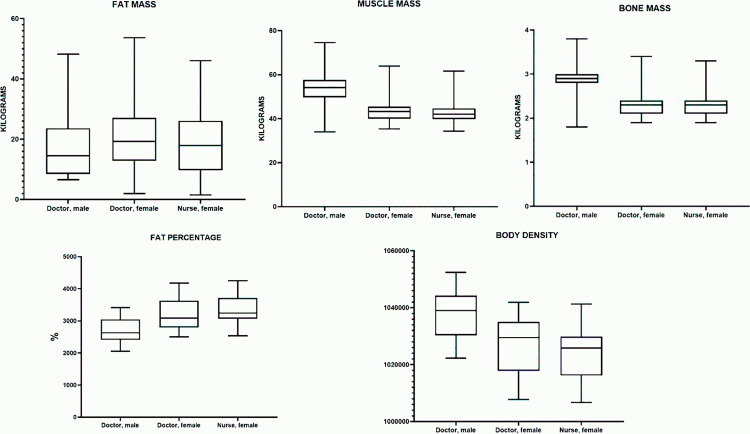
Body density, fat percentage, muscle mass, bone mass, and fat mass in the studied groups.

Male physicians statistically differed significantly in body density from both female groups (with female physicians having a 6.5% higher value, p = 0.0007, and female nurses having a 1.2% lower value, p < 0.0001, compared to male physicians). However, the differences between female physicians and female nurses were statistically insignificant (Fig 3).

A comparison between groups using the non-parametric Kruskal–Wallis test followed by Dunn’s post-hoc test revealed statistically significant differences between: “male physicians” and “female physicians” (p = 0.0007), “male physicians” and “female nurses” (p < 0.0001). At the same time, the differences between “female physicians” and “female nurses” were statistically insignificant (p = 0.1459). Thus, male physicians significantly differed in body density from both female groups, whereas no statistically significant differences were found between female physicians and female nurses.

#### Fat percentage indicators.

The median body fat percentage (Me) in the male physician group was 26.33% (24.08–30.46%), in the female physician group – 30.85% (27.97–36.30%), and in the female nurse group – 32.47% (30.67–37.11%). Thus, the highest median body fat percentage was recorded in female nurses, followed by female physicians, while male physicians had the lowest values. The interquartile range (IQR) was also wider in the female physician and female nurse groups, indicating greater variability in fat percentage within these groups. (Table 3).

The fat percentage in male physicians was significantly lower than in both female groups: in female physicians, it was higher by 15.6% (p = 0.0006), and in female nurses, it was higher by 32.2% (p < 0.0001) compared to male physicians. At the same time, the difference of approximately 14% (p = 0.1667) between female physicians and female nurses did not reach statistical significance (Fig 3). Comparison between groups using the Kruskal–Wallis test followed by Dunn’s test showed statistically significant differences between the groups: “male physicians” and “female physicians” (p = 0.0006), “male physicians” and “female nurses” (p < 0.0001). However, differences between the groups “female physicians” and “female nurses” did not reach statistical significance (p = 0.1667). Thus, the fat percentage in male physicians was significantly lower than in both female groups, while no statistically significant differences were observed between female physicians and female nurses.

#### Muscle mass.

The median muscle mass (Me) in the male physician group was 54.20 kg (49.68–57.65 kg), in the female physician group – 43.25 kg (39.90–45.43 kg), and in the female nurse group – 42.00 kg (39.80–44.70 kg). Thus, the highest median muscle mass was observed in male physicians, while the lowest was in female nurses. The interquartile range was wider in the male physician group, indicating greater variability in muscle mass within this category. (Table 3).

In terms of percentage differences in median values (relative to male physicians), female physicians had a 21.1% lower value (p = 0.0007), and female nurses had an 18.1% lower value (p < 0.0001). Meanwhile, the difference of approximately 3.8% between female physicians and female nurses was statistically insignificant (p = 0.9119) (Fig 3).

Comparison between groups using the Kruskal–Wallis test followed by Dunn’s test revealed statistically significant differences between the groups: “male physicians” and “female physicians” (p = 0.0007), “male physicians” and “female nurses” (p < 0.0001), while differences between “female physicians” and “female nurses” (p = 0.9119) did not reach statistical significance. Thus, muscle mass in male physicians was significantly higher than in both female groups, while no statistically significant differences were found between female physicians and female nurses.

#### Bone mass.

The median bone mass (Me) in the male physician group was 2.900 kg (2.800–3.000 kg), in the female physician group – 2.300 kg (2.100–2.400 kg), and in the female nurse group – 2.300 kg (2.100–2.400 kg). The highest median bone mass was recorded in male physicians, while the interquartile range was wider in this group, indicating greater variability in bone mass among male physicians. (Table 3).

In terms of percentage differences (relative to male physicians), female physicians had a 17.9% lower median bone mass (p < 0.0001), and female nurses also had a 17.9% lower median bone mass (p < 0.0001). Meanwhile, the difference between female physicians and female nurses was statistically insignificant (p > 0.9999) (Fig 3).

Comparison between groups using the Kruskal–Wallis test followed by Dunn’s test showed statistically significant differences between the groups “male physicians” and “female physicians” (p < 0.0001), “male physicians” and “female nurses” (p < 0.0001), while differences between “female physicians” and “female nurses” (p > 0.9999) did not reach the level of statistical significance. Thus, bone mass in male physicians was significantly higher than in both female groups, while no statistically significant differences were found between female physicians and female nurses.

#### Fat mass.

The median fat mass (Me) in the male physician group was 14.50 kg (8.45–23.65 kg), in the female physician group – 19.20 kg (12.80–27.05 kg), and in the female nurse group – 17.90 kg (9.70–26.10 kg). The highest median fat mass was observed in female physicians, while the male physician group exhibited the widest interquartile range, indicating greater variability in fat mass among male physicians. (Table 3).

Regarding the relative difference in medians, the fat mass in female physicians was approximately 40% higher than in male physicians, but this difference was not statistically significant (p = 0.4931). In female nurses, it was more than twice as high (approximately 100% or more) compared to male physicians, but this difference was also not statistically significant (p > 0.9999). The difference of approximately 46% between female physicians and female nurses also did not reach statistical significance (p = 0.5570) (Fig 3).

Comparison between groups using the Kruskal–Wallis test followed by Dunn’s test showed that in all pairwise comparisons (p > 0.05), the differences were not statistically significant. Thus, despite a visually higher median fat mass in the female groups, the difference did not reach statistical significance. Ultimately, no significant differences in fat mass were identified among the three studied groups (male physicians, female physicians, and female nurses).

### Characteristics of actual food intake (macronutrients)

#### Proteins.

All groups showed differences in protein intake relative to the recommended norm. In male physicians, the median intake was 66.46 g, which is 11.4% below the recommended norm (75.0 g) and is not statistically significant (p = 0.0684). In female physicians, the median protein intake was 53.52 g, which is 4.4% below the recommended norm (56.0 g) and is not statistically significant (p = 0.6471). A similar situation was observed in female nurses, where the median intake was 54.11 g, 3.4% below the recommended norm (56.0 g), and also not statistically significant (p = 0.9114). Thus, all groups showed a slight decrease in protein intake relative to the norm, but the differences did not reach statistical significance.

The median dietary protein intake (Me) in the male physician group was 66.46 g (44.90–80.49 g), in the female physician group – 53.52 g (35.09–72.93 g), and in the female nurse group – 54.11 g (34.28–74.91 g). The highest median protein intake was observed in male physicians, while the female nurse group exhibited the widest interquartile range, indicating greater variability in protein consumption. (Table 4).

**Table 4:** Characteristics of actual dietary intake (macronutrients) in the studied groups.

Indicator	Male physicians	Female physicians	Female nurses
	Me (25%–75%)	Me (25%–75%)	Me (25%–75%)
**Protein (g)**	66.46 (44.90–80.49)	53.52 (35.09–72.93)	54.11 (34.28–74.91)
**Lipids (g)**	84.93 (49.82–134.40)	57.85 (42.44–95.20)	58.22 (40.58–98.56)
**Carbohydrates (g)**	268.8 (211.3–326.8)	199.5 (144.4–288.1)	211.2 (153.4–277.5)
**Dietary Fiber (g)**	13.81 (9.283–16.50)	12.70 (7.884–16.80)	11.35 (8.205–15.96)
**Caloric Intake (kcal)**	2180 (968.3–2437)	1613 (1227–2137)	1666 (1216–2264)

**Fig 4:**
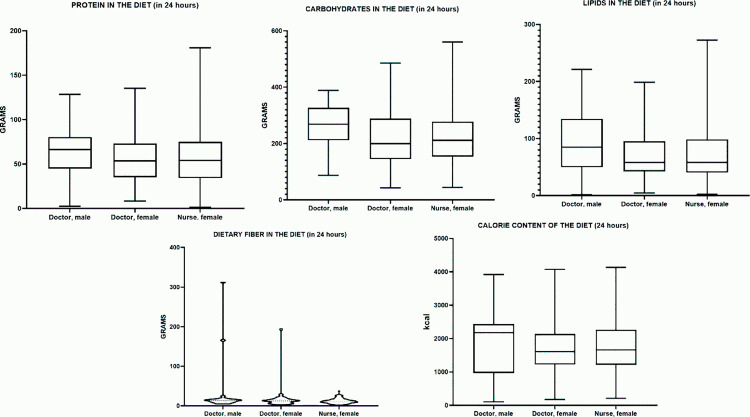
Characteristics of actual food intake (macronutrients) in the studied groups.

In terms of percentage differences (relative to male physicians), the median protein intake in female physicians was approximately 20% lower (p = 0.9800), and in female nurses, it was 19% lower (p = 0.9440). Meanwhile, the difference between female physicians and female nurses (~1%) was not statistically significant (p > 0.9999) (Fig 4).

Comparison between groups using the Kruskal–Wallis test and Dunn’s post hoc test showed no statistically significant differences in any pairwise comparisons (p > 0.05). Thus, protein intake among the three studied groups (male physicians, female physicians, and female nurses) does not differ significantly.

#### Lipids.

All groups showed significant differences in lipid intake relative to the recommended norm. In male physicians, the median lipid intake was 84.93 g, which is 15.9% above the recommended lipid intake norm (73.30 g); however, this difference was not statistically significant (p = 0.1055). In female physicians, the median lipid intake was 57.85 g, which is 5.2% above the recommended lipid intake norm (55.00 g) and was statistically significant (p = 0.0253). A similar situation was observed in female nurses, where the median lipid intake was 58.22 g, 5.9% above the recommended norm (55.00 g) and also statistically significant (p = 0.0016). Thus, male physicians exhibited a tendency toward exceeding the recommended lipid intake norm, but without statistical significance, whereas female physicians and nurses significantly exceeded the norm, as confirmed by the high statistical significance of the differences.

The median dietary lipid intake (Me) in the male physician group was 84.93 g (49.82–134.40 g), in the female physician group – 57.85 g (42.44–95.20 g), and in the female nurse group – 58.22 g (40.58–98.56 g). The highest median lipid intake was observed in male physicians, while the female nurse group exhibited the widest interquartile range, indicating greater variability in lipid consumption. (Table 4).

In terms of percentage differences (relative to male physicians), the median lipid intake in female physicians was approximately 32% lower (p = 0.1364), and in female nurses, it was 31% lower (p = 0.1331). The difference between female physicians and female nurses (~1%) was also statistically insignificant (p > 0.9999) (Fig 4).

Comparison between groups using the Kruskal–Wallis test with Dunn’s post hoc test did not reveal significant differences (p > 0.05). Thus, lipid intake in the studied groups does not differ statistically significantly.

#### Carbohydrates.

All groups showed significant differences in carbohydrate intake relative to the recommended norm. In male physicians, the median carbohydrate intake was 268.8 g, which is 18.6% below the recommended carbohydrate intake norm (330.0 g) and is statistically significant (p = 0.0032). In female physicians, the median carbohydrate intake was 199.5 g, which is 19.2% below the recommended norm (247.0 g) and is statistically significant (p = 0.0434). A similar situation was observed in female nurses, where the median intake was 211.2 g, 14.5% below the recommended norm (247.0 g), and also statistically significant (p = 0.0015). Thus, all groups demonstrated insufficient carbohydrate intake, which is confirmed by the high statistical significance of the differences.

The median dietary carbohydrate intake (Me) in the male physician group was 268.8 g (211.3–326.8 g), in the female physician group – 199.5 g (144.4–288.1 g), and in the female nurse group – 211.2 g (153.4–277.5 g). The highest median carbohydrate intake was observed in male physicians, while the female nurse group exhibited the widest interquartile range, indicating greater variability in carbohydrate consumption (Table 4).

In terms of percentage differences (relative to male physicians), the median carbohydrate intake in female physicians was approximately 6% higher (p = 0.1475), and in female nurses, it was also 6% higher (p = 0.1217). Differences between female physicians and female nurses were nearly absent and statistically insignificant (p > 0.9999) (Fig 4). Comparison using the Kruskal–Wallis test followed by Dunn’s test confirmed the absence of statistically significant differences (p > 0.05) in all pairwise comparisons. Thus, carbohydrate intake in male physicians, female physicians, and female nurses does not show significant differences.

#### Dietary fiber.

All groups showed differences in dietary fiber intake relative to the recommended norm. In male physicians, the median dietary fiber intake was 13.81 g, which is 63.7% below the recommended dietary fiber intake norm (38 g/day), and this difference is statistically significant (p = 0.0022). In female physicians, the median dietary fiber intake was 17.93 g, which is 28.3% below the recommended norm (25 g/day); however, the statistical significance of this difference was absent (p = 0.0632). In female nurses, the median dietary fiber intake was 12.43 g, which is 50.3% below the recommended norm (25 g/day) and is statistically significant (p < 0.0001). Thus, all groups, especially male physicians and female nurses, consumed dietary fiber in insufficient amounts, with the statistical significance of differences being most pronounced in male physicians and female nurses.

The median dietary fiber intake (Me) in the male physician group was 13.81 g (9.283–16.50 g), in the female physician group – 12.70 g (7.884–16.80 g), and in the female nurse group – 11.35 g (8.205–15.96 g). The highest median fiber intake was recorded in male physicians, while the female physician group showed a wider interquartile range, indicating greater variability in fiber consumption (Table 4).

In terms of percentage differences (relative to male physicians), the median dietary fiber intake in female physicians was approximately 8% lower (p > 0.9999), and in female nurses, it was 18% lower (p = 0.5030). Meanwhile, the difference between female physicians and female nurses (~10%) also did not reach statistical significance (p = 0.9853) (Fig 4). Comparison between groups using the Kruskal–Wallis test with Dunn’s test showed no significant differences (p > 0.05) between any pairs. Thus, dietary fiber intake does not demonstrate significant differences based on gender or professional status.

#### Caloric intake.

All groups showed significant differences in calorie intake relative to the recommended norm. In male physicians, the median caloric intake was 2180 kcal, which is 9.2% below the recommended caloric intake norm (2400 kcal) and is statistically significant (p = 0.0393). In female physicians, the median caloric intake was 1613 kcal, which is 10.4% below the recommended norm (1800 kcal) and is not statistically significant (p = 0.1302). A similar situation was observed in female nurses, where the median caloric intake was 1666 kcal, 7.4% below the recommended norm (1800 kcal), and also not statistically significant (p = 0.3256). Thus, male physicians demonstrated a statistically significant reduction in caloric intake compared to the recommended norm, whereas in female physicians and nurses, this deficit was less pronounced and did not reach statistical significance.

The median daily caloric intake (Me) in the male physician group was 2180 kcal (968.3–2437 kcal), in the female physician group – 1613 kcal (1227–2137 kcal), and in the female nurse group – 1666 kcal (1216–2264 kcal). The highest median caloric intake was observed in male physicians, while female physicians exhibited a wider interquartile range, suggesting greater variability in energy consumption (Fig 4).

Comparison between groups using the Kruskal–Wallis test and Dunn’s post hoc test did not reveal any significant differences (p > 0.05).

Thus, neither gender nor professional affiliation has a statistically significant impact on caloric intake. In all three groups (male physicians, female physicians, and female nurses), actual energy consumption remained below the recommended ranges (2400–2800 kcal for men and 1800–2200 kcal for women), indicating a general trend toward caloric deficit among the study participants.

### Mineral intake

#### Iron.

All groups showed significant differences in iron intake relative to the recommended norm. In male physicians, the median iron intake was 12.12 mg, which is 51.5% above the recommended iron intake (8.00 mg) and is statistically significant (p = 0.0003). In female physicians, the median iron intake was 11.91 mg, which is 33.9% below the recommended norm (18.00 mg) and is statistically significant (p < 0.0001). A similar situation was observed in female nurses, where the median iron intake was 11.74 mg, 34.8% below the recommended norm (18.00 mg), and also statistically significant (p < 0.0001). Thus, male physicians significantly exceed the iron intake norm, whereas female physicians and nurses experience a substantial iron deficiency, confirmed by the high statistical significance of the differences.

The median daily iron intake (Me) in the male physician group was 12.12 mg (9.055–16.17 mg), in the female physician group – 11.91 mg (9.605–14.35 mg), and in the female nurse group – 11.74 mg (8.850–15.29 mg). The highest median iron intake was observed in male physicians, while female nurses exhibited a slightly lower median value. However, the interquartile range was widest in male physicians, indicating greater variability in iron intake within this group (Table 5).

**Table 5:** Characteristics of mineral intake in the studied groups.

Indicator	Male doctors	Female doctors	Female nurses
	Me (25%–75%)	Me (25%–75%)	Me (25%–75%)
**Iron (Fe, mg)**	12.12 (9.055–16.17)	11.91 (9.605–14.35)	11.74 (8.850–15.29)
**Potassium (K, mg)**	1599 (1214–2437)	1786 (1540–2473)	1728 (1191–2122)
**Calcium (Ca, mg)**	378.3 (242.0–521.1)	375.9 (271.4–589.2)	435.6 (317.7–583.7)
**Magnesium (Mg, mg)**	180.9 (125.4–262.6)	191.0 (146.3–266.9)	194.2 (141.4–246.1)
**Sodium (Na, mg)**	3131 (2085–4580)	2670 (1714–4206)	2609 (1856–4228)
**Phosphorus (P, mg)**	922.0 (622.0–1087)	800.7 (638.1–1039)	808.1 (585.4–1095)

**Fig 5:**
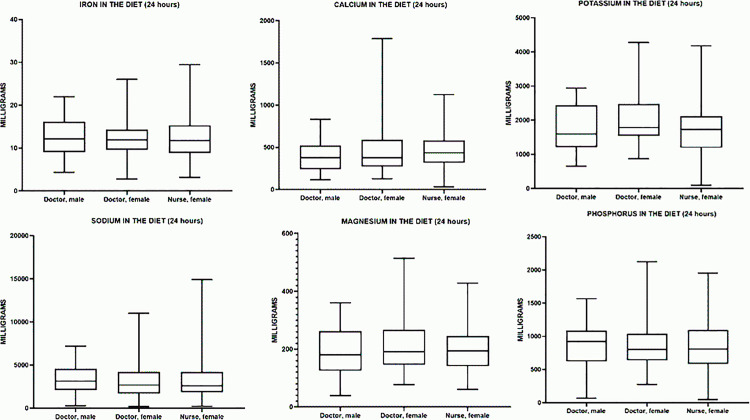
Comparative analysis of median daily mineral intake in the studied groups.

In terms of percentage differences (relative to male physicians), the median iron intake in female physicians was approximately 2% lower (p > 0.9999), and in female nurses, it was 3% lower (p > 0.9999). The difference between female physicians and female nurses (~1%) was also statistically insignificant (p > 0.9999) (Fig 5). Comparison between groups using the Kruskal–Wallis test and Dunn’s post hoc test showed no statistically significant differences in any pairwise comparisons (p > 0.05). Thus, iron intake (or levels) among the three studied groups (male physicians, female physicians, and female nurses) does not differ significantly.

#### Potassium.

All groups showed significant differences in potassium intake relative to the recommended norm (4700 mg/day). In male physicians, the median potassium intake was 1599 mg, which is 66.0% below the recommended potassium intake, and this difference is statistically significant (p < 0.0001). In female physicians, the median potassium intake was 1786 mg, which is 61.9% below the recommended norm and is also statistically significant (p < 0.0001). A similar situation was observed in female nurses, where the median potassium intake was 1728 mg, 63.2% below the recommended norm, and this difference was also statistically significant (p < 0.0001). Thus, all groups showed substantial potassium deficiency, confirmed by the statistical significance of the differences.

The median daily potassium intake (Me) in the male physician group was 1599 mg (1214–2437 mg), in the female physician group – 1786 mg (1540–2473 mg), and in the female nurse group – 1728 mg (1191–2122 mg). The highest median potassium intake was observed in female physicians, while female nurses exhibited a slightly lower median value. However, the interquartile range was widest in male physicians, indicating greater variability in potassium intake within this group (Table 5).

In terms of percentage differences (relative to male physicians), the median potassium intake in female physicians was approximately 12% higher (p > 0.9999), and in female nurses, it was 8% higher (p > 0.9999). The difference between female physicians and female nurses (~3%) was also statistically insignificant (p = 0.2120) (Fig 5). Comparison between groups using the Kruskal–Wallis test and Dunn’s post hoc test showed no statistically significant differences in any pairwise comparisons (p > 0.05). Thus, potassium intake (or levels) among the three studied groups (male physicians, female physicians, and female nurses) does not differ significantly.

#### Calcium.

All groups showed significant differences in calcium intake relative to the recommended norm (1000 mg/day). In male physicians, the median calcium intake was 378.3 mg, which is 62.2% below the recommended intake, and this difference is statistically significant (p < 0.0001). In female physicians, the median calcium intake was 375.9 mg, which is 62.4% below the recommended intake and also statistically significant (p < 0.0001). A similar situation was observed in female nurses, where the median calcium intake was 435.6 mg, 56.4% below the recommended intake, and this difference was also statistically significant (p < 0.0001). Thus, all groups showed substantial calcium deficiency, confirmed by the statistical significance of the differences.

The median daily calcium intake (Me) in the male physician group was 378.3 mg (242.0–521.1 mg), in the female physician group – 375.9 mg (271.4–589.2 mg), and in the female nurse group – 435.6 mg (317.7–583.7 mg). The highest median calcium intake was observed in female nurses, while male and female physicians had similar median values. However, the interquartile range was wider in female physicians, suggesting greater variability in calcium intake within this group (Table 5).

In terms of percentage differences (relative to male physicians), the median calcium intake in female physicians was 0.6% lower (p > 0.9999), while in female nurses, it was 15% higher (p = 0.2549). Meanwhile, the difference between female physicians and female nurses (~16%) was statistically insignificant (p = 0.7041) (Fig 5). Comparison between groups using the Kruskal–Wallis test and Dunn’s post hoc test showed no statistically significant differences in any pairwise comparisons (p > 0.05). Thus, calcium intake (or levels) among the three studied groups (male physicians, female physicians, and female nurses) does not differ significantly.

#### Magnesium.

All groups showed significant differences in magnesium intake relative to the recommended norm (420 mg/day for men, 310 mg/day for women). In male physicians, the median magnesium intake was 180.9 mg, which is 56.9% below the recommended intake, and this difference is statistically significant (p < 0.0001). In female physicians, the median magnesium intake was 191.0 mg, which is 38.4% below the recommended intake and also statistically significant (p < 0.0001). A similar situation was observed in female nurses, where the median magnesium intake was 194.2 mg, 37.4% below the recommended intake, and this difference was also statistically significant (p < 0.0001). Thus, all groups showed substantial magnesium deficiency, confirmed by the statistical significance of the differences.

The median daily magnesium intake (Me) in the male physician group was 180.9 mg (125.4–262.6 mg), in the female physician group – 191.0 mg (146.3–266.9 mg), and in the female nurse group – 194.2 mg (141.4–246.1 mg). The highest median magnesium intake was observed in female nurses, while male physicians had the lowest median value. However, the interquartile range was wider in female physicians, indicating greater variability in magnesium intake within this group (Table 5).

In terms of percentage differences (relative to male physicians), the median magnesium intake in female physicians was approximately 10% higher (p > 0.9999), in female nurses – approximately 5% lower (p > 0.9999); the difference between female physicians and female nurses (~15%) was also statistically insignificant (p > 0.9999) (Fig 5).

Comparison between groups using the Kruskal–Wallis test and Dunn’s post hoc test showed no significant differences in any pairwise comparisons (p > 0.05). Thus, magnesium intake in the three studied groups does not differ significantly.

#### Sodium.

All groups showed significant differences in sodium intake relative to the recommended norm (1500 mg/day). In male physicians, the median sodium intake was 3131 mg, which is 108.7% above the recommended intake, and this difference is statistically significant (p < 0.0001). In female physicians, the median sodium intake was 2670 mg, which is 78.0% above the recommended intake and also statistically significant (p < 0.0001). A similar situation was observed in female nurses, where the median sodium intake was 2609 mg, 73.9% above the recommended intake, and this difference was also statistically significant (p < 0.0001). Thus, all groups significantly exceeded the recommended sodium intake level, confirmed by the statistical significance of the differences.

The median daily sodium intake (Me) in the male physician group was 3131 mg (2085–4580 mg), in the female physician group – 2670 mg (1714–4206 mg), and in the female nurse group – 2609 mg (1856–4228 mg). The highest median sodium intake was observed in male physicians, while the lowest was recorded in female nurses. However, the widest interquartile range was seen in male physicians, suggesting greater variability in sodium intake within this group (Table 5).

In terms of percentage differences (relative to male physicians), the median sodium intake in female physicians was approximately 10% lower (p > 0.9999), in female nurses – 15% lower (p > 0.9999); the difference between female physicians and female nurses (~5%) was also statistically insignificant (p > 0.9999) (Fig 5). The Kruskal–Wallis test and Dunn’s post hoc test did not reveal significant intergroup differences (p > 0.05). Thus, sodium intake in the three studied groups is statistically comparable.

#### Phosphorus.

All groups showed significant differences in phosphorus intake relative to the recommended norm (1000 mg/day). In male physicians, the median phosphorus intake was 922 mg, which is 7.8% below the recommended intake; however, this difference was not statistically significant (p = 0.1465). In female physicians, the median phosphorus intake was 800.7 mg, which is 19.9% below the recommended intake and is statistically significant (p < 0.0001). A similar situation was observed in female nurses, where the median phosphorus intake was 808.1 mg, 19.2% below the recommended intake, and this difference was also statistically significant (p < 0.0001). Thus, female physicians and nurses showed a substantial phosphorus deficiency, confirmed by the statistical significance of the differences, while the reduction in phosphorus intake in male physicians was not statistically significant.

The median daily phosphorus intake (Me) in the male physician group was 922.0 mg (622.0–1087 mg), in the female physician group – 800.7 mg (638.1–1039 mg), and in the female nurse group – 808.1 mg (585.4–1095 mg). The highest median phosphorus intake was observed in male physicians, while the lowest was recorded in female physicians. However, the widest interquartile range was seen in female nurses, suggesting greater variability in phosphorus intake within this group (Table 5).

In terms of percentage differences (relative to male physicians), the median phosphorus intake in female physicians was approximately 5% higher (p > 0.9999), in female nurses – ~ 10% lower (p > 0.9999); the difference between female physicians and female nurses (~15%) also did not reach statistical significance (p > 0.9999) (Fig 5). Comparison between groups using the Kruskal–Wallis test (p > 0.05) and Dunn’s post hoc analysis showed no significant differences in any pairwise comparisons. Thus, phosphorus intake among male physicians, female physicians, and female nurses does not differ significantly.

### Vitamin intake

#### Thiamine (Vitamin B1).

All groups showed significant differences in thiamine (vitamin B1) intake relative to the recommended norm (1.1 mg/day). In male physicians, the median thiamine intake was 0.7498 mg, which is 31.9% below the recommended intake, and this difference is statistically significant (p < 0.0001). In female physicians, the median thiamine intake was 0.575 mg, which is 47.7% below the recommended intake and also statistically significant (p < 0.0001). A similar situation was observed in female nurses, where the median thiamine intake was 0.5738 mg, 47.8% below the recommended intake, and this difference was also statistically significant (p < 0.0001). Thus, all groups showed a substantial deficiency in thiamine intake, confirmed by the statistical significance of the differences.

The median daily thiamine (vitamin B₁) intake (Me) in the male physician group was 0.7498 mg (0.4988–0.9311 mg), in the female physician group – 0.5750 mg (0.4480–0.8178 mg), and in the female nurse group – 0.5738 mg (0.4241–0.7948 mg). The highest median thiamine intake was observed in male physicians, while the lowest was recorded in female nurses. However, the widest interquartile range was seen in male physicians, suggesting greater variability in thiamine intake within this group (Table 6).

**Table 6:** Characteristics of water-soluble vitamin intake in the studied groups.

Indicator	Male Doctors	Female Doctors	Female Nurses
	Me (25%–75%)	Me (25%–75%)	Me (25%–75%)
**Vitamin B1** **(Thiamine, mg)**	0.749 (0.498–0.931)	0.575 (0.448–0.817)	0.573 (0.424–0.794)
**Vitamin B2** **(Riboflavin, mg)**	0.814 (0.646–1.214)	0.812 (0.570–1.046)	0.8108 (0.599–1.077)
**Vitamin C (mg)**	22.44 (12.45–39.12)	25.55 (12.74–53.35)	18.61 (8.668–31.72)
**Vitamin PP** **(Niacin, mg)**	9.934 (7.868–16.17)	11.39 (5.865–14.70)	9.088 (5.043–13.92)

**Fig 6:**
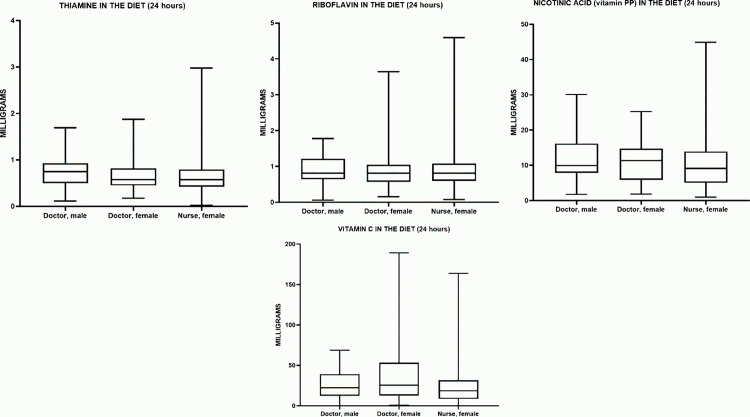
Characteristics of water-soluble vitamin intake in the studied groups.

In terms of percentage differences (relative to male physicians), the median thiamine intake in female physicians was approximately 23% lower (p = 0.5956), and in female nurses, it was 24% lower (p = 0.3261). The difference between female physicians and female nurses (~0.2%) was statistically insignificant (p > 0.9999) (Fig 6). Comparison between groups using the Kruskal–Wallis test and Dunn’s post hoc test showed no statistically significant differences in any pairwise comparisons (p > 0.05). Thus, thiamine (B1) intake (or levels) among the three studied groups (male physicians, female physicians, and female nurses) does not differ significantly.

#### Riboflavin (Vitamin B2).

All groups showed significant differences in riboflavin (vitamin B2) intake relative to the recommended norm (1.3 mg/day). In male physicians, the median riboflavin intake was 0.8148 mg, which is 37.3% below the recommended intake, and this difference is statistically significant (p = 0.0003). In female physicians, the median riboflavin intake was 0.8125 mg, which is 37.5% below the recommended intake and also statistically significant (p < 0.0001). A similar situation was observed in female nurses, where the median riboflavin intake was 0.8108 mg, 37.6% below the recommended intake, and this difference was also statistically significant (p < 0.0001). Thus, all groups showed a substantial riboflavin deficiency, confirmed by the statistical significance of the differences.

The median daily riboflavin (vitamin B₂) intake (Me) in the male physician group was 0.8148 mg (0.6460–1.214 mg), in the female physician group – 0.8125 mg (0.5700–1.046 mg), and in the female nurse group – 0.8108 mg (0.5991–1.077 mg). The highest median riboflavin intake was observed in male physicians, while the lowest was recorded in female physicians. However, the widest interquartile range was seen in male physicians, suggesting greater variability in riboflavin intake within this group (Table 6).

In terms of percentage differences (relative to male physicians), the median riboflavin intake in female physicians was approximately 0.3% lower (p > 0.9999), and in female nurses, it was 0.5% lower (p > 0.9999). The difference between female physicians and female nurses (~0.2%) was also statistically insignificant (p > 0.9999) (Fig 6). Comparison between groups using the Kruskal–Wallis test and Dunn’s post hoc test showed no statistically significant differences in any pairwise comparisons (p > 0.05). Thus, riboflavin (B2) intake (or levels) among the three studied groups (male physicians, female physicians, and female nurses) does not differ significantly.

#### Vitamin PP (Niacin).

All groups showed significant differences in niacin (vitamin PP) intake relative to the recommended norm (16 mg/day for men and 14 mg/day for women). In male physicians, the median niacin intake was 9.934 mg, which is 37.9% below the recommended intake, and this difference is statistically significant (p = 0.0059). In female physicians, the median niacin intake was 11.39 mg, which is 18.6% below the recommended intake and also statistically significant (p = 0.0010). A similar situation was observed in female nurses, where the median niacin intake was 9.088 mg, 35.1% below the recommended intake, and this difference was also statistically significant (p < 0.0001). Thus, all groups showed a deficiency in niacin intake, confirmed by the statistical significance of the differences.

The median daily niacin (vitamin PP) intake (Me) in the male physician group was 9.934 mg (7.868–16.17 mg), in the female physician group – 11.39 mg (5.865–14.70 mg), and in the female nurse group – 9.088 mg (5.043–13.92 mg). The highest median niacin intake was observed in female physicians, while the lowest was recorded in female nurses. However, the widest interquartile range was seen in male physicians, suggesting greater variability in niacin intake within this group (Table 6).

In terms of percentage differences (relative to male physicians), the median niacin intake in female physicians was approximately 15% higher (p > 0.9999), and in female nurses, it was approximately 9% lower (p = 0.5581). The difference between female physicians and female nurses (~20%) was also statistically insignificant (p = 0.5760) (Fig 6). Comparison between groups using the Kruskal–Wallis test and Dunn’s post hoc test showed no statistically significant differences in any pairwise comparisons (p > 0.05). Thus, niacin (vitamin PP) intake among the three studied groups (male physicians, female physicians, and female nurses) does not differ significantly.

#### Vitamin C.

All groups showed significant differences in vitamin C intake relative to the recommended norm (90 mg/day for men and 75 mg/day for women). In male physicians, the median vitamin C intake was 22.44 mg, which is 74.5% below the recommended intake, and this difference is statistically significant (p < 0.0001). In female physicians, the median vitamin C intake was 25.55 mg, which is 66.0% below the recommended intake and also statistically significant (p < 0.0001). A similar situation was observed in female nurses, where the median vitamin C intake was 18.61 mg, 75.2% below the recommended intake, and this difference was also statistically significant (p < 0.0001). Thus, all groups showed a substantial deficiency in vitamin C intake, confirmed by the statistical significance of the differences.

The median daily vitamin C intake (Me) in the male physician group was 22.44 mg (12.45–39.12 mg), in the female physician group – 25.55 mg (12.74–53.35 mg), and in the female nurse group – 18.61 mg (8.668–31.72 mg). The highest median vitamin C intake was observed in female physicians, while the lowest was recorded in female nurses. However, the widest interquartile range was seen in female physicians, suggesting greater variability in vitamin C intake within this group (Table 6).

In terms of percentage differences (relative to male physicians), the median vitamin C intake in female physicians was approximately 14% higher (p > 0.9999), and in female nurses, it was approximately 17% lower (p > 0.9999). The difference between female physicians and female nurses (~27%) was statistically significant (p = 0.0378) (Fig 6).

Comparison between groups using the Kruskal–Wallis test and Dunn’s post hoc test revealed a significant difference only between female physicians and female nurses (p < 0.05). Thus, vitamin C intake among male physicians, female physicians, and female nurses is generally similar, except for the pair “female physicians – female nurses,” where a statistically significant difference was found.

## Discussion

Our study conducted a detailed analysis of the anthropometric parameters and nutritional status of healthcare professionals (male physicians, female physicians, and nurses) to assess the extent to which sex and professional status influence age, body composition, and the intake of key macro- and micronutrients.

Regarding chronological age, male physicians exhibited slightly higher values compared to the female groups. However, statistically significant differences were observed only between male physicians and nurses, with male physicians being older. No significant differences were found between male and female physicians or between female physicians and nurses. In terms of metabolic age, no substantial differences were detected among the three groups. Although men and women may have different metabolic levels nominally, these groups did not differ at a statistically significant level.

In all groups, metabolic age was noticeably lower than chronological age, reflecting a relatively satisfactory body composition and, on average, an absence of pronounced obesity among participants. The greatest “reserve of youth” in metabolic parameters was observed in male physicians despite their higher chronological age. Differences between the two female groups (physicians and nurses) were minimal in this regard, indicating similar trends in the proportion of muscle, fat, and bone tissues. Since metabolic age largely reflects body composition and basal metabolic rate, our results confirm the absence of pronounced obesity that could “accelerate aging.” These findings align with previous studies showing that healthcare professionals often maintain relatively stable body weight indicators but may experience hidden deficiencies in certain nutrients [32,33].

Analysis of body weight showed that male physicians were, on average, heavier than nurses, and this difference was statistically significant. No significant differences were found between male and female physicians or between female physicians and nurses, although median values varied slightly. When considering body mass index (BMI), none of the comparisons between groups revealed statistically significant differences. This suggests that, despite noticeable variations in body weight or age, the weight-to-height ratio remains comparable across all three groups. Similar BMI results among healthcare professionals, without clear differences based on sex or professional role, were previously reported in a study of surgeons in Bishkek, which identified an issue of unbalanced nutrition but no evident obesity [34].

The study of body density showed that male physicians differed significantly from both female physicians and nurses. However, no statistically significant differences were found between the two female groups. In terms of fat percentage, male physicians had lower overall values compared to female physicians and nurses, although the differences between the female groups did not reach statistical significance. Similarly, muscle mass analysis showed that male physicians had significantly higher median values than both female groups, while no significant differences were observed between female physicians and nurses. Finally, bone mass was also higher in male physicians compared to both female groups. These findings correspond to known physiological sex differences. A study by Sum (2024) noted that work schedules and shift types may have an impact, but height and weight parameters are primarily influenced by sex rather than professional role [35].

Thus, in terms of parameters such as age, body weight, body density, muscle mass, and bone mass, male physicians generally exhibited higher values, while females, regardless of whether they were physicians or nurses, displayed a higher fat percentage (despite similar BMI values). Similar trends have been recorded in other studies, which note that in various medical institutions, men have greater muscle mass, whereas female groups exhibit different body composition characteristics [36,37].

No statistically significant differences were found between female physicians and nurses in any of the analyzed anthropometric parameters. However, male physicians sometimes exhibited statistically significant differences compared to nurses (e.g., in body weight and age) and, in some cases, compared to female physicians (e.g., in body density, muscle mass, and bone mass). This suggests that sex is a more significant factor than professional role, which aligns with findings from studies on the nutrition of surgeons and outpatient clinic staff, where sex-related differences were more pronounced than those attributed to professional activity [38,39].

Although all groups exceeded the recommended fat intake in absolute terms, only the female physicians and nurses showed a statistically significant difference from the norm. Analysis of fat intake demonstrated that all groups significantly exceeded the upper acceptable limits. Carbohydrate intake was generally insufficient, and dietary fiber consumption was also well below the necessary level. This problematic dietary profile – characterized by low fiber intake, excess fats, and protein deficiency – has been noted in other studies on physician nutrition, where the primary causes were cited as lack of time and irregular eating schedules [40,41].

The identified patterns in macronutrient intake did not reveal statistically significant differences between men and women or between female physicians and nurses. This finding aligns with previous studies indicating that the eating habits of healthcare professionals are primarily determined by shift schedules and the lack of accessible food infrastructure rather than sex or specific professional roles [42,43].

The intake of several minerals (iron, potassium, calcium, magnesium, sodium, and phosphorus) in all three groups either did not reach the recommended level (iron in women; calcium, potassium, magnesium, and phosphorus in all groups) or exceeded it (sodium in all groups). The observed shortfall of iron in female physicians and nurses (≈ 35% below RDA) raises concern about latent iron deficiency anemia, a condition that can diminish physical stamina, reduce cognitive performance, and increase fatigue—factors that directly affect clinical efficiency. Conversely, male physicians consumed ≈ 12 mg day - ¹, exceeding the male RDA but remaining far below the UL of 45 mg day - ¹; therefore, the risk of iron overload disorders appears minimal.

However, statistical tests did not reveal significant differences by sex or professional status. This result supports conclusions that “objective magnesium and potassium deficiency can be observed in both physicians and nurses regardless of work schedule,” as previously noted in multiple studies [28,43]. It also reinforces the view that surgeons, therapists, and nurses often face the same issues related to mineral deficiencies [33].

Regarding vitamin PP, vitamin C, riboflavin (B₂), and thiamine (B₁), a pronounced deficiency of these nutrients was identified in all groups. However, statistically significant differences were observed only for vitamin C within female groups: female physicians had slightly higher levels than nurses. Several studies emphasize that vitamin deficiencies are particularly prevalent among female healthcare professionals due to the combination of professional and household responsibilities, as well as overall workload [35,44]. However, our study indicates that male physicians may also experience similar vitamin deficiencies, without a clear advantage in this regard.

This finding is consistent with previous studies indicating that therapists and nurses often exhibit similar unfavorable dietary habits, primarily due to time constraints and the lack of structured meal organization (31). In particular, social and organizational barriers such as short meal breaks and a limited selection of available foods equally affect all categories of healthcare professionals [45,46].

## Limitations

This study has a descriptive cross-sectional design, which does not allow for the establishment of causal relationships. We can only identify associations and trends rather than prove that observed dietary deficiencies are the cause or consequence of specific factors.

The study was conducted in six clinics within a single city in Central Kazakhstan (Karaganda), which limits the generalizability of the findings to other regions of Kazakhstan and, even more so, to other Central Asian countries.

Not all categories of healthcare professionals were included in sufficient numbers (e.g., male nurses were excluded due to their low representation), which limits the ability to compare certain subgroups.

A single 24-hour dietary recall may not accurately reflect the typical diet. Participants may have provided inaccurate data due to forgetfulness, conscious or unconscious response adjustments (social desirability bias), or the fact that the survey day may not have been representative in terms of meal frequency and portion sizes.

Researchers did not intervene in the habitual diets of respondents and had no opportunity to monitor them in real-time (e.g., through photo diaries or mobile applications). This limitation increases the risk of self-report biases.

Participation in the study was voluntary, which may have led to a self-selection bias, where individuals more concerned about their health were more likely to participate. This could result in an overestimation of favorable dietary patterns.

The assessment of vitamin and mineral intake was based solely on dietary intake data and bioelectrical impedance analysis, without biochemical blood tests (if such tests were not conducted). As a result, the actual nutrient status may differ from the estimated values. We did not collect haematological indices (e.g., haemoglobin, ferritin) or data on blood‑donation frequency; consequently, we could not verify clinical anaemia in females or evaluate potential iron overload/ donation practices in males.

In some subgroups (e.g., male physicians), the relatively small number of participants may have led to wider confidence intervals and reduced statistical power in detecting intergroup differences.

Although data collection took place from 2023 to 2024, dietary intake was assessed during specific periods. Seasonal variations (availability of fresh vegetables and fruits, changes in eating habits across different seasons) may not have been fully accounted for.

We did not record specific chronic metabolic or cardiovascular diagnoses; consequently, residual confounding by medically prescribed diets cannot be fully ruled out, although no participant reported following a restrictive diet during the 24‑h recalls.

## Conclusions

Primary‑care physicians and nurses showed widespread deficits in protein, fibre, calcium, potassium, magnesium, phosphorus and several B‑group vitamins, together with excess sodium and fat intake. Sex or professional role exerted little influence on dietary patterns, although male physicians had higher muscle and bone mass. The pervasive nutrient inadequacy highlights an institutional need for scheduled meal breaks and healthier food options. Addressing these gaps could improve staff well‑being and patient care quality.

### Practical significance

1. The obtained data can serve as a foundation for developing targeted programs to improve the nutritional status of physicians and nurses in clinics. This is particularly relevant for reducing long-term risks of chronic non-communicable diseases (obesity, cardiovascular diseases, osteoporosis, etc.).
2. The study results indicate that even professionally informed healthcare workers experience deficiencies in key vitamins and minerals. Therefore, it is advisable to revise hospital cafeteria menus, introduce balanced meals rich in vegetables, fruits, sources of “slow” carbohydrates, and dietary fiber.
3. Based on the findings, informational and educational materials on rational nutrition can be prepared for PHC personnel, including visual guides on balanced diets to provide clear references for protein, fat, carbohydrate, vitamin, and mineral intake.
4. Improving the nutritional status of healthcare personnel may positively impact professional performance, stress resistance, and burnout prevention, ultimately influencing the quality of patient care.
5. The results allow for the formulation of hypotheses for larger multicenter studies with a longitudinal design and the expansion of geographical coverage beyond the region. This would help assess whether the identified issues are also prevalent among healthcare workers in other regions of Kazakhstan and neighboring Central Asian countries.

## Supporting information

S1 Appendix Informed consent of the patient. Questionnaire.
(PDF)
