# Supplementary material for: Nutritional status of healthcare professionals in primary health and social care
Source: PLoS One. 2025 Jun 4;20(6):e0325422. doi: 10.1371/journal.pone.0325422 (PMC12136426; doi:10.1371/journal.pone.0325422)
Supplement: S1 Appendix — Questionnaire. (PDF) [file pone.0325422.s001.pdf]

Ministry of Healthcare of the Republic of Kazakhstan
Karaganda Medical University

Research participant questionnaire
"Assessment of the actual nutrition status of medical workers in Karaganda"

Registration number _____________
Region of residence________________________

City____________________________________

District_________________________________

Karaganda 2023

Information for the research participant

Dear respondent,

We invite you to participate in the program: *" Nutritional status of healthcare professionals in primary health and social care."*

The purpose of this study is to conduct a detailed comparative analysis of the actual nutrition status of primary healthcare (PHC) medical workers in Karaganda, considering differences between doctors and mid-level medical personnel.

The assessment of actual nutrition status will be conducted using standardized international questionnaires.

The study is conducted with the voluntary informed consent of the respondent, and no financial compensation is provided for participation in the survey. All data obtained from respondents will be kept confidential.

Dear respondent, we kindly ask you to answer the questions in the questionnaire. If you agree to participate in this study, you need to read and sign the informed consent form.

Thank you for your cooperation!

Informed consent of the respondent

I have been thoroughly and clearly informed about the scientific study *" Nutritional status of healthcare professionals in primary health and social care."*

I hereby voluntarily agree, without compensation, to participate in this scientific study. I understand that I will be asked to fill out the provided questionnaires or respond to interview questions. I have been clearly informed that any information obtained through this survey is confidential. During data processing, my data will be anonymized (making participant identification impossible) and will be used exclusively for scientific purposes.

I also consent to undergo bioimpedance analysis and skinfold measurements using a caliper as part of this research. I acknowledge that these procedures are noninvasive, pose minimal risk, and will be performed following established scientific and ethical guidelines.

Full name:

____

Address:

____

Contact phone number: ____________________________

(*This information is required by the organizers for possible clarification of your questionnaire responses, remains confidential, and will not be used elsewhere.*)

Date: "_____" ______________ 2023

Signature: ______________

Assessment of the actual nutrition status of the elderly population

1.1 Place of residence (specify city, region, village, etc.)

1.2 Date of questionnaire completion ________________________________

1.3 Age/Date of birth _________________________________

1.4 Gender
Male Female (underline the appropriate option)

1.5 Marital status

- Married or living in a civil partnership - Divorced or living separately
- Never married
- Widower/Widow
- Difficult to answer
- Refused to answer

1.6 What is your level of education?

- Below secondary Secondary
- Secondary specialized - Higher (including incomplete higher education, if 3 years or more)
- Difficult to answer
- Refused to answer

1.7 What is the total household income? (Total amount including all sources: salary, pension, benefits, and other payments)

- Less than 50,000 tenge
- 50,000 - 100,000 tenge
- 100,000 - 200,000 tenge
- More than 200,000 tenge

2. Anthropometry

2.1 What is your weight? _______________ kg

2.2 What is your height? _______________ cm

2.3 Skinfold thickness

____
____
____
____
____

3. Awareness of the principles of healthy eating

3.1 How much fresh vegetables and fruits do you think should be consumed daily?
- Amount in grams ______________ g
- Difficult to answer ______________
- Refused to answer ______________

3.2 Which foods should predominate in the daily diet? Rate the products on a 5-point scale: from 1 to 5, where 1 – the product should be present in the largest amount in the diet, 5 – in the smallest amount
- Fats, sweets ___________________
- Meat and fish products ___________
- Milk and dairy products ___________
- Vegetables, fruits ________________
- Grains, bread, potatoes ____________
- Difficult to answer ______________
- Refused to answer ______________

3.3 In your opinion, which type of milk is the most beneficial for health?
- Low-fat or fat-free (fat content 2.5%-0.5%) ____
- Regular milk with a fat content of about 3.2% or higher (6%)
- Difficult to answer ___________________________________
- Refused to answer __________________________________

3.4 What type of salt do you think is best to consume?
- None
- Regular salt
- Iodized salt
- Difficult to answer
- Refused to answer

3.5 From a health perspective, which fat is best for cooking?
- No fat
- Vegetable oil
- Margarine
- Butter
- Any type of fat
- Difficult to answer
- Refused to answer

3.6 Where do you primarily get information on proper nutrition?
- Medical workers
- Books, brochures
- Relatives, acquaintances
- Mass media
- Other, specify _______________________________________
- Difficult to answer
- Refused to answer

4. Eating habits

4.1 What type of fat do you use for cooking?

- Do not use
- Vegetable oil
- Margarine
- Butter
- All types of fat
- Difficult to answer

4.2 What type of fat do you most often use for sandwiches?

- Do not use butter or margarine
- Margarine
- Butter
- Difficult to answer
- Refused to answer

4.3 What fat content of milk do you usually drink?

- I do not drink milk
- Low-fat or fat-free (fat content 0.5%-2.5%)
- Regular milk with a fat content of about 3.2% or higher (6%)
- Difficult to answer
- Refused to answer

4.4 Is there always a choice of dairy products with different fat contents in your store?

- Rarely or never
- Sometimes
- Always
- Difficult to answer
- Refused to answer

4.5 Do you usually add extra salt to your food at the table?

- Never
- Yes, if it is not salty enough
- I almost always add salt without tasting it
- Difficult to answer
- Refused to answer

4.6 How often do you use iodized salt?

- Rarely or never
- Sometimes
- Always
- Difficult to answer
- Refused to answer

4.7 How often do you consume the following foods? (*For each product, select only one answer*)

№	Products	Every day	Several times a week	Once a week	Several times a month	No more than once a month	Rarely or never
1	Liver						
2	Sausages, hot dogs						
3	Meat (beef, pork, poultry)						
4	Fish and seafood						
5	Milk and dairy products						
6	Potatoes						
7	Vegetables (excluding potatoes)						
8	Fruits, berries						
9	Grains, pasta						
10	Sweets (candies, jam, honey)						
11	Bread and bakery products						
12	Eggs						

5. Daily food diary

Please describe what you ate and drank over the past 24 hours, from the moment you woke up in the morning until the time you went to bed. If you ate or drank during the night, please include that as well. Do not forget to mention any food or drinks consumed outside your home. Include all types of food and beverages you consumed. Also, specify where you ate and where the food was prepared.

5.1 Please tell us, yesterday you ate:

- The same amount as usual - 1
- Less than usual - 2
- More than usual - 3
- Difficult to answer - 7
- Refused to answer - 9

5.2 Was your food intake yesterday related to...

- **Doctor's recommendations**
  - Yes - 1
  - No - 2
  - Difficult to answer - 7
  - Refused to answer - 9
- **Following a special diet**
  - Yes - 1
  - No - 2
  - Difficult to answer - 7
  - Refused to answer - 9
- **Religious practices**
  - Yes - 1
  - No - 2
  - Difficult to answer - 7
  - Refused to answer - 9

5.3 Indicate how you ate yesterday:

№	Meal time	Place of eating	Name and composition of the product, dish, or drink	Preparation	Amount (g/ml)	CODE
1st meal		Home (as a guest)-1 Enterprise Canteen-2 School, kindergarten, etc.-3 Workplace-4 Other-5				
2nd meal		Home (as a guest)-1 Enterprise Canteen-2 School, kindergarten, etc.-3 Workplace-4 Other-5				
3rd meal		Home (as a guest)-1 Enterprise Canteen-2 School, kindergarten, etc.-3 Workplace-4 Other-5				
4th meal		Home (as a guest)-1 Enterprise Canteen-2 School, kindergarten, etc.-3 Workplace-4 Other-5				
5th meal		Home (as a guest)-1 Enterprise Canteen-2 School, kindergarten, etc.-3 Workplace-4 Other-5				
6th meal		Home (as a guest)-1 Enterprise Canteen-2 School, kindergarten, etc.-3 Workplace-4 Other-5				
7th meal		Home (as a guest)-1 Enterprise Canteen-2 School, kindergarten, etc.-3 Workplace-4 Other-5				
8th meal		Home (as a guest)-1 Enterprise Canteen-2 School, kindergarten, etc.-3 Workplace-4 Other-5				
9th meal		Home (as a guest)-1 Enterprise Canteen-2 School, kindergarten, etc.-3 Workplace-4 Other-5				

Interviewer's comments *(Please provide your comments on this questionnaire)*

I certify that the survey was conducted by me in accordance with the Instructions using the personal interview method with the respondent selected according to the Instructions.

Signature ______________
